# Complete Genome Sequence of a Classical Swine Fever Virus Isolate Belonging to New Subgenotype 2.1d from Henan Province, Central China

**DOI:** 10.1128/genomeA.00093-16

**Published:** 2016-05-12

**Authors:** Chaochao Lv, Qingyuan Yang, Xiaojing Gao, Yali Yao, Xiangdong Li, Yan Xiao, Kegong Tian

**Affiliations:** a National Research Center for Veterinary Medicine, High-Tech District, Luoyang, People’s Republic of China; b College of Animal Science and Veterinary Medicine, Henan Agricultural University, Zhengzhou, People’s Republic of China

## Abstract

We report here the complete genome sequence of HeN1505, a field isolate of classical swine fever virus belonging to the new subgenotype 2.1d. HeN1505 distinguishes itself from other classical swine fever virus (CSFVs) by 1 amino acid substitution in position 159 (threonine by isoleucine), which led to the loss of one *N*-glycosylation site in the N^pro^ protein.

## GENOME ANNOUNCEMENT

Classical swine fever (CSF) is one of the Office International des Epizooties (OIE) notifiable diseases (1). Classical swine fever virus (CSFV), the causative agent of CSF, is a single-stranded positive RNA virus of the genus *Pestivirus* within the family *Flaviviridae* (2). The CSFV genome is approximately 12.3 kb in length, and it comprises a single long open reading frame (ORF) that encodes a polyprotein that is co- and posttranslationally cleaved to form mature structural and nonstructural proteins (3). Phylogenetic analysis based on the E2 gene and a partial nonstructural 5B (NS5B) gene divides CSFV into three genotypes (1–3), with each being further divided into three or four subgenotypes (1.1 to 1.3, 2.1 to 2.3, and 3.1 to 3.4) (4). CSFV subgenotype 2.1 has been further divided into 2.1a, 2.1b, and 2.1c (5, 6). In mainland China, CSFVs of subgenotype 2.1b have predominated since the 1990s (7, 8). Recently, Zhang et al. (9) reported a new subgenotype, 2.1d.

Here, we describe the complete genome sequence of a field isolate of CSFV, HeN1505, which was isolated from a pig farm in Henan Province, China, in 2015. The full nucleotide sequence was obtained from seven overlapping fragments amplified by reverse transcription-PCR and was assembled and manually edited to produce the final genome sequence. The complete genome sequence of isolate HeN1505 is 12,296 nucleotides (nt) in length, with a 5′ untranslated region (UTR) of 373 nt and a 3′ UTR of 226 nt. A single large ORF is 11,697 nt between nucleotide positions 374 and 12070 and is capable of coding for a polyprotein of 3,898 amino acids.

Phylogenetic analysis based on the genome and full-length E2 gene indicated that CSFV HeN1505 was located in the new subgenotype 2.1d, which was shifting away from historical and vaccine strains in both phylogenetic trees. CSFV HeN1505 was most closely related to a subgenotype 2.1d strain, JSZL, reported recently by Zhang et al. (10), with 1.9% and 2.1% nucleotide sequence differences in the genome and full-length E2 gene, respectively. More nucleotide differences were found between the full-length E2 gene of HeN1505 and those of the subgenotypes 2.1a, 2.1 b, 2.1c, and 2.1d  CSFVs, at 6.0 to 6.7%, 3.7 to 6.0%, 9.1 to 9.3%, and 1.1 to 3.7%, respectively. Compared with subgenotypes 2.1a, 2.1b, and 2.1c, subgenotype 2.1d  isolates, including HeN1505, have 1 amino acid substitution at position 34 (N^34^S) in the E2 protein. Compared with other subgenotypes of CSFV, subgenotype 2.1d isolates, including HeN1505, have 2 amino acid substitutions (GGK/R→GRI at positions 2 and 3, and F/V^40^L) in the NS2 protein. The unique genetic marker for HeN1505 is 1 amino acid substitution at position 159 (threonine by isoleucine) compared with all the other CSFV sequences available in GenBank, which leads to a loss of a potential *N*-glycosylation site in the N^pro^ protein. The above data provide valuable information on the molecular epidemiology of CSFV and facilitate investigation on the genetic evolution of CSFV field strains in China.

### Nucleotide sequence accession number.

The complete genome sequence of CSFV HeN1505 has been deposited in GenBank under the accession no. KU556758.
